# Beyond identity categories: chronotype and mental health as correlates of sleep quality in transgender and gender-diverse adults

**DOI:** 10.3389/fpsyt.2026.1872006

**Published:** 2026-07-03

**Authors:** Paolo Meneguzzo, Alessio A. Gugliotta, Angela Favaro, Sara Montagnese, Marina Bonato, Alberto Scala, Marina Miscioscia, Andrea Garolla

**Affiliations:** 1 Department of Neuroscience, University of Padova, Padova, Italy; 2 Padova Neuroscience Center, University of Padova, Padova, Italy; 3 Regional Reference Center for Gender Incongruence, Azienda Ospedale-Università di Padova, Padova, Italy; 4 Department of Medicine, University of Padova, Padova, Italy; 5 Department of Developmental Psychology and Socialization, University of Padova, Padova, Italy; 6 Unit of Andrology and Reproductive Medicine, Department of Medicine, University of Padova, Padova, Italy

**Keywords:** chronotype, circadian preference, gender-affirming hormone therapy, mental health, sleep quality, transgender and gender-diverse

## Abstract

**Background:**

Sleep disturbance is frequently reported among transgender and gender-diverse (TGD) adults, yet the relative contribution of circadian preference, mental health, and gender-affirming hormone therapy (GAHT) remains unclear.

**Methods:**

In this cross-sectional study, 222 TGD adults (mean age = 26.9 ± 9.3 years; 15.3% non-binary) attending a specialized gender clinic in Padova (Italy) completed the Pittsburgh Sleep Quality Index (PSQI), Morningness–Eveningness Questionnaire (MEQ), and SF-12. Multivariable linear regression examined associations between sleep quality and chronotype, mental health-related quality of life, age, sex assigned at birth, gender identity, and current GAHT. An exploratory model tested the interaction between chronotype and GAHT status.

**Results:**

Mean PSQI was 6.31 ± 3.84; 54.9% scored >5 and 31.5% ≥8. Poorer sleep quality was independently associated with greater eveningness, lower mental health-related quality of life, younger age, and current GAHT. The association between eveningness and poorer sleep quality was stronger among participants receiving GAHT. Within the GAHT subgroup, treatment duration was not associated with PSQI scores.

**Conclusions:**

Poor sleep quality is common in TGD adults and appears primarily associated with circadian preference and psychological well-being. Findings suggest that the relationship between chronotype and sleep quality may vary by GAHT status, although no linear association with treatment duration was observed. Longitudinal studies are needed to clarify these relationships.

## Highlights

- Poor sleep quality is highly prevalent among transgender and gender-diverse adults, with substantial variability across individuals.
- Chronotype and mental health-related quality of life are the strongest correlates of sleep quality in this population.
- Eveningness is associated with poorer sleep quality, particularly among individuals receiving gender-affirming hormone therapy.
- No association was found between duration of hormone therapy and sleep quality, suggesting a non-linear relationship.
- Sleep disturbances in TGD populations may reflect multidimensional vulnerability rather than identity-specific effects.

## Introduction

Sleep quality is a central component of psychological well-being and everyday functioning across the general population (1). Variations in sleep continuity, timing, and subjective quality are closely intertwined with emotional regulation, cognitive performance, and perceived health, and have therefore gained increasing attention in psychiatric research as dimensional correlates of mental health rather than disorder-specific symptoms (2–4).

Within this framework, poor sleep quality has been conceptualized as a transdiagnostic indicator of psychological burden, reflecting the interaction between affective processes, circadian regulation, and environmental demands. Rather than representing a discrete clinical condition, sleep disturbance is increasingly viewed as a clinically meaningful phenotype that cuts across diagnostic boundaries and varies along a continuum in both clinical and non-clinical populations (2, 3). Importantly, sleep quality also shows well-established sex differences in the general population, with women consistently reporting greater sleep disturbance and a higher prevalence of insomnia symptoms than men, suggesting that sex-related biological and psychosocial factors may contribute to variability in sleep outcomes (5).

Transgender and gender-diverse (TGD) individuals are people whose gender identity differs, fully or partially, from the sex assigned at birth, including binary transgender and non-binary identities. Although prevalence estimates vary according to definitions, sampling methods, and age groups, recent studies suggest that approximately 0.5–1% of adults identify as transgender or gender-diverse, with higher estimates among younger populations (6, 7). These populations have been increasingly included in sleep and mental health research, reflecting broader efforts to understand health-related experiences across diverse populations (8, 9). Available evidence suggests that sleep difficulties are commonly reported in TGD samples, often alongside elevated levels of psychological distress and reduced health-related quality of life (7, 10). Sleep disparities in TGD populations are unlikely to be explained by a single mechanism. Existing literature suggests a multifactorial framework in which biological factors, psychological burden, and social stressors converge. On the one hand, transgender populations appear to report high rates of poor sleep, insomnia symptoms, and related impairment in quality of life. On the other hand, broader sexual and gender minority sleep research suggests that sleep disturbance may also reflect chronic exposure to minority stress, including stigma, discrimination, social rejection, and anticipatory vigilance. Within this perspective, sleep problems in TGD individuals may be understood not simply as isolated symptoms, but as clinically relevant indicators of broader dysregulation at the intersection of circadian, affective, and social processes (11–13). Minority stress theory is particularly relevant in this context (14). According to this framework, individuals belonging to stigmatized minority groups are exposed to chronic stressors, including discrimination, prejudice, expectations of rejection, concealment, and internalized stigma, which accumulate over time and negatively affect physical and mental health. In sexual and gender minority populations, these processes have been linked to sustained activation of stress systems, psychological distress, and poorer sleep quality. This model is consistent with the view that identity-related variables do not act directly on sleep in a simple categorical manner; rather, their effects may be mediated through psychological distress, hyperarousal, and environmental adversity. For TGD populations, this framework is especially useful because it allows sleep difficulties to be interpreted in relation to lived context, rather than as intrinsic features of gender identity itself (12). However, the factors associated with sleep quality in this population remain incompletely understood, and existing findings highlight substantial heterogeneity rather than uniform patterns of impairment.

Chronotype, defined as an individual preference for the timing of sleep and wakefulness, represents a relatively stable, biologically grounded trait (15, 16). Eveningness has been associated with emotional dysregulation, mood and anxiety symptoms, and poorer subjective sleep quality in both clinical and non-clinical samples (15–17). Importantly, chronotype is increasingly conceptualized as an individual-level vulnerability factor that may interact with psychological burden to influence sleep-related outcomes, rather than as a categorical feature distinguishing specific groups. Chronotype may represent one of the key individual-level dimensions through which vulnerability to sleep disturbance is expressed. Eveningness has been repeatedly associated with poorer subjective sleep, worse mood, and reduced quality of life, and may therefore function as a trait-like vulnerability factor rather than as a group-defining characteristic. This distinction is clinically important, because it suggests that circadian preference may help explain heterogeneity within TGD samples even when identity categories alone do not (18).

Interest in circadian functioning within TGD populations has recently expanded, particularly in relation to gender-affirming hormone therapy (GAHT). Chronotype may be especially relevant in this population because sleep difficulties are common, psychological distress is elevated compared with the general population, and both factors have been consistently linked to eveningness. Furthermore, recent evidence suggests that sex hormones may influence circadian timing, raising the possibility that biological and psychosocial factors unique to TGD populations may interact with individual circadian preference. Prospective evidence indicates that sex hormones may induce modest shifts in circadian timing (19), raising questions about the extent to which sleep disturbances observed in TGD adults reflect treatment-related changes versus broader circadian and psychological vulnerability. At the same time, cross-sectional studies report inconsistent findings regarding differences by sex assigned at birth, gender identity, or treatment status (9, 11, 18).

Despite increasing research on sleep in TGD populations, the field remains fragmented. Some studies have focused on descriptive differences in sleep burden, others on associations with mental health or quality of life, and more recent work has begun to examine the possible role of GAHT. However, these dimensions have rarely been considered together within the same multivariable framework. In particular, it remains unclear whether sleep quality in TGD adults is more strongly associated with individual circadian preference and current psychological well-being than with identity-related or treatment-related characteristics.

The present study was designed to examine sleep quality in a clinically recruited sample of transgender and gender-diverse adults and to investigate its associations with chronotype, mental health-related quality of life, and GAHT. Unlike previous studies, which have primarily focused on descriptive sleep outcomes or single correlates of sleep health, the present study considered circadian preference, psychological well-being, identity-related variables, and treatment-related factors within the same multivariable framework.

We hypothesized that poorer sleep quality would be associated with greater eveningness and lower mental health-related quality of life. Given the inconsistent literature regarding the role of sex assigned at birth, gender identity, and GAHT, analyses involving these variables were considered exploratory. Finally, we explored whether the association between chronotype and sleep quality differed according to current GAHT status.

## Methods

### Participants

This cross-sectional study included TGD adults attending the Regional Reference Center for Gender Incongruence at the Padova University Hospital, a multidisciplinary outpatient service providing assessment and gender-affirming care. Participants were consecutively recruited during routine clinical assessments. Inclusion criteria were age ≥18 years and the ability to complete self-report questionnaires. Participation was voluntary and no financial or other incentives were provided. Participation was voluntary and no financial or other incentives were provided.

Sociodemographic and clinical information, including age, sex assigned at birth (AMAB/AFAB), gender identity (binary vs. non-binary), and GAHT status, were collected during the clinical assessment. For participants receiving GAHT, treatment duration was recorded in months. The final sample consisted of 222 participants.

The study was approved by the Comitato Etico Territoriale Area Centro-Est Veneto (CET-ACEV; approval code CEV-6011/AO/24) and conducted in accordance with the Declaration of Helsinki. All participants provided written informed consent prior to participation.

### Measures

#### Sleep quality

Sleep quality was assessed using the Pittsburgh Sleep Quality Index (PSQI), a widely used self-report questionnaire evaluating subjective sleep quality over the previous month (20, 21). The PSQI yields a global score ranging from 0 to 21, with higher scores indicating poorer sleep quality. Sleep quality, indexed by the PSQI total score, was considered the primary outcome of the study. In addition to continuous scores, established PSQI cut-offs (>5 and ≥8) were used descriptively to characterize the distribution and severity of sleep disturbance, but not as inferential outcomes (22).

#### Chronotype

Chronotype was assessed using the Morningness–Eveningness Questionnaire (MEQ), which measures individual preference for the timing of sleep and wakefulness (23, 24). Higher scores indicate greater morningness, while lower scores reflect eveningness. Chronotype was primarily analyzed as a continuous variable to maximize statistical power. Standard MEQ categories (morning, intermediate, and evening types) were additionally used descriptively to facilitate clinical interpretation.

#### Mental health–related quality of life

Mental health–related quality of life was assessed using the Mental Component Summary (MCS) of the 12-Item Short Form Health Survey (SF-12) (25, 26). Higher MCS scores indicate better perceived mental health and functioning. The SF-12 MCS was included as an indicator of psychological burden.

### Statistical analysis

Descriptive statistics were used to summarize sociodemographic and clinical characteristics of the sample. Continuous variables were reported as means and standard deviations or medians and interquartile ranges, as appropriate, whereas categorical variables were reported as frequencies and percentages.

The primary analysis consisted of a multivariable linear regression model with PSQI total score as the dependent variable. Age, chronotype (MEQ total score), mental health-related quality of life (SF-12 MCS), current GAHT (yes/no), sex assigned at birth (AMAB vs. AFAB), and gender identity (binary vs. non-binary) were included as independent variables. To further examine whether the association between chronotype and sleep quality differed according to hormone treatment status, an additional exploratory model included the interaction term between MEQ total score and current GAHT status. Given the cross-sectional design, this analysis was intended to characterize patterns of association rather than to test treatment effects.

A secondary exploratory analysis was then conducted only among participants receiving GAHT, with treatment duration (months) entered as an independent variable in place of GAHT status, to examine whether longer exposure was associated with PSQI scores. PSQI cut-offs (>5 and ≥8) were used only descriptively and were not treated as inferential outcomes. Additional exploratory analyses were conducted to examine potential differences in sleep quality and chronotype according to sex assigned at birth and gender identity. These analyses were intended to provide descriptive context and were not considered primary study outcomes.

All statistical tests were two-tailed, with a significance threshold set at p <.05. Analyses were performed using IBM SPSS Statistics version 25 (IBM Corp., Armonk, NY).

## Results

The final sample consisted of 222 transgender and gender-diverse adults. The mean age was 26.9 years (SD = 9.3). Thirty-four participants (15.3%) identified as non-binary, while 188 (84.7%) identified as binary. At the time of assessment, 100 participants (45.0%) were receiving GAHT. Descriptive characteristics of the sample are reported in **Table 1**.

**Table 1:** Sociodemographic and clinical characteristics of the sample, overall and by current GAHT status.

Variable	Total sample (N = 222)	No GAHT (n = 122)	Current GAHT (n = 100)
Age, years	26.90 ± 9.28	25.80 ± 9.53	28.24 ± 8.86
BMI, kg/m²	23.35 ± 5.75	23.55 ± 5.32	23.12 ± 6.27
Sex assigned at birth, AMAB	95 (42.8%)	46 (38.0%)	49 (49.0%)
Non-binary gender identity	34 (15.3%)	23 (19.0%)	11 (11.0%)
MEQ total score	43.37 ± 9.82	42.72 ± 9.79	44.16 ± 9.89
SF-12 Mental Component Summary (MCS)	46.97 ± 8.54	45.58 ± 7.75	48.80 ± 9.04
PSQI total score, mean ± SD	6.31 ± 3.84	5.63 ± 3.48	7.06 ± 4.07
PSQI total score, median [IQR]	6.0 [3.0–8.0]	5.0 [3.0–8.0]	6.0 [4.0–11.0]
PSQI > 5	122 (55.0%)	59 (48.8%)	62 (62.0%)
PSQI ≥ 8	70 (31.5%)	32 (26.4%)	37 (37.0%)
GAHT duration, months, median [IQR]*	–	–	1.0 [0.8–11.2]
Chronotype: extreme evening	23 (10.4%)	13 (10.7%)	10 (10.0%)
Chronotype: moderate evening	94 (42.3%)	54 (44.6%)	40 (40.0%)
Chronotype: intermediate	92 (41.4%)	43 (35.5%)	48 (48.0%)
Chronotype: moderate morning	13 (5.9%)	11 (9.1%)	2 (2.0%)
Chronotype: extreme morning	0 (0.0%)	0 (0.0%)	0 (0.0%)

Values are presented as mean ± standard deviation unless otherwise specified. AMAB, assigned male at birth; BMI, body mass index; GAHT, gender-affirming hormone therapy; IQR, interquartile range; MEQ, Morningness-Eveningness Questionnaire; MCS, Mental Component Summary; PSQI, Pittsburgh Sleep Quality Index. *Reported only for participants receiving current GAHT.

### Sleep quality

Overall sleep quality was poor. The mean PSQI total score was 6.31 (SD = 3.84), with a median of 6.0 [IQR: 3.0–8.0]. Using established thresholds, 54.9% (n = 122) of participants scored above the traditional PSQI >5 cut-off, while 31.5% (n = 70) met a more conservative criterion for clinically relevant sleep disturbance (PSQI ≥8).

### Chronotype

Chronotype scores indicated a predominance of intermediate-to-evening preference in the sample. The mean MEQ score was 43.4 (SD = 9.8). When categorized, 42.3% of participants were classified as moderately evening type, 41.4% as intermediate type, 10.4% as extreme evening type, and 5.9% as moderately morning type. No participants were classified as extreme morning type. Additional exploratory analyses were conducted according to sex assigned at birth and gender identity. Mean PSQI scores did not differ significantly between AFAB and AMAB participants (5.94 ± 3.96 vs. 6.79 ± 3.63, p = .104), nor did MEQ scores (44.08 ± 9.96 vs. 42.42 ± 9.59, p = .217). In contrast, significant differences emerged across gender identity groups. Non-binary participants reported higher PSQI scores (8.10 ± 4.42) than transgender men (5.84 ± 3.91) and transgender women (6.26 ± 3.34) (F = 4.19, p = .016). Similarly, non-binary participants showed lower MEQ scores, indicating greater eveningness (38.50 ± 9.94), compared with transgender men (44.34 ± 9.90) and transgender women (43.86 ± 9.26) (F = 4.46, p = .013). No significant differences in chronotype distribution were observed according to current GAHT status.

### Primary multivariable analysis: GAHT status

A multivariable linear regression analysis was conducted in the total sample. The overall model was statistically significant and explained approximately 26% of the variance in PSQI scores (adjusted R² = .26). Poorer sleep quality was independently associated with greater eveningness (MEQ: B = −0.13, p <.001), lower mental health-related quality of life (MCS: B = −0.13, p <.001), younger age (B = −0.06, p = .015), and current GAHT status (B = 2.24, p <.001), whereas sex assigned at birth and gender identity were not independently associated with PSQI scores.

To clarify the role of GAHT, an additional exploratory model tested the interaction between chronotype and current GAHT status. This model explained a larger proportion of variance in PSQI scores (adjusted R² = .36). The interaction term between MEQ and GAHT was statistically significant (B = −0.26, p <.001), indicating that the association between greater eveningness and poorer sleep quality was stronger among participants receiving GAHT. In this model, lower MCS and younger age remained independently associated with poorer sleep quality, whereas sex assigned at birth and gender identity were not significant.

**Figure 1:**
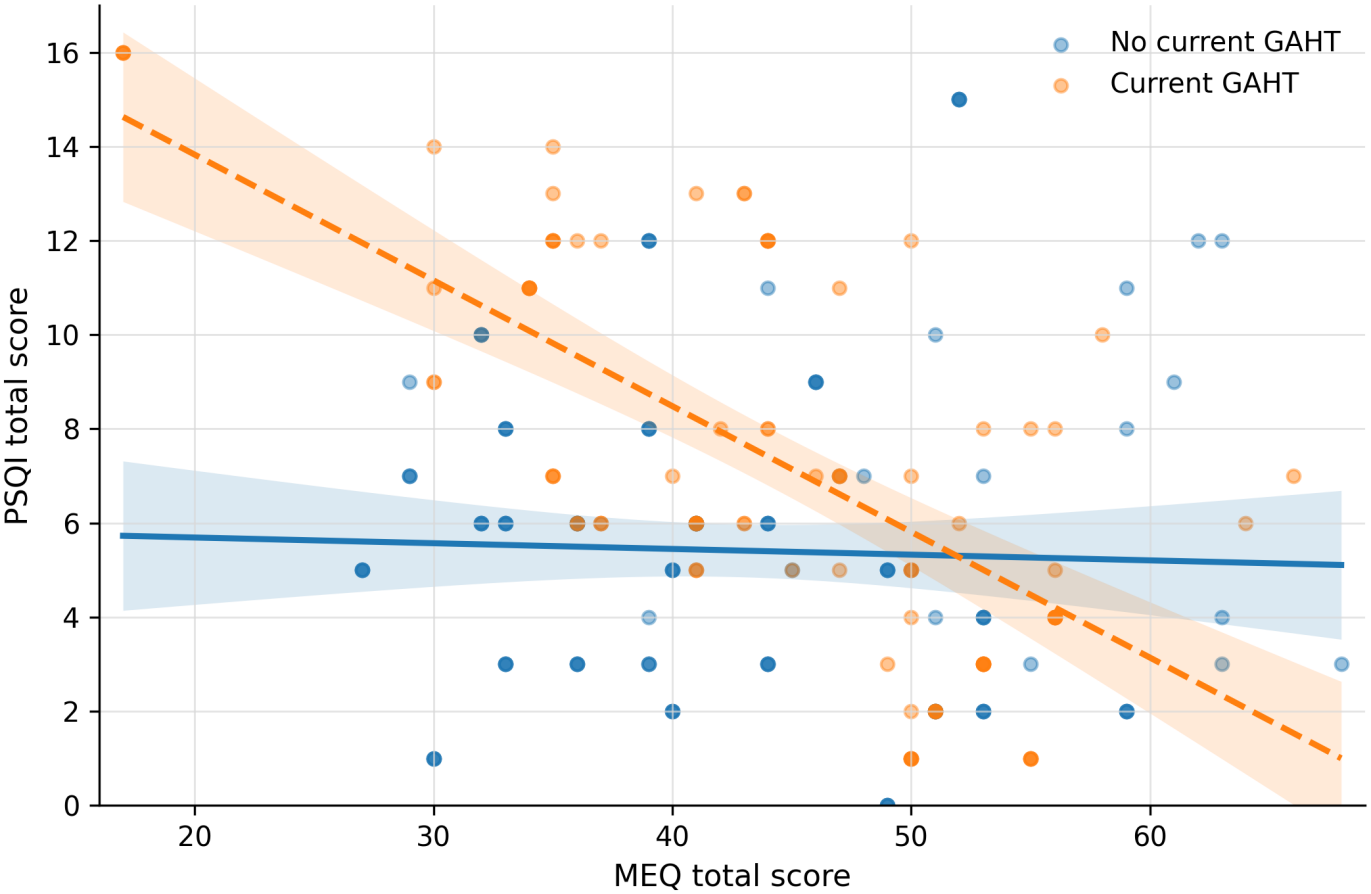
Adjusted association between chronotype (MEQ total score) and sleep quality (PSQI total score) according to current gender-affirming hormone therapy (GAHT) status. Solid and dashed lines represent model-based predicted PSQI scores from the multivariable regression including the MEQ × GAHT interaction, adjusted for age, mental health-related quality of life, sex assigned at birth, and binary versus non-binary gender identity. Shaded areas indicate 95% confidence intervals. Points represent observed individual data.

Consistent with this interaction, the association between chronotype and sleep quality was weak and not statistically significant among participants not receiving GAHT, whereas it was stronger among those receiving GAHT. These subgroup patterns are descriptive and should not be interpreted as evidence of a treatment effect. This pattern is illustrated in **Figure 1**.

### Secondary analysis: treatment duration

A secondary exploratory multivariable linear regression was conducted among participants receiving GAHT to examine whether treatment duration was associated with sleep quality. The overall model was statistically significant (F(6,93) = 25.35, p <.001) and explained a substantial proportion of variance in PSQI scores (R² = .62; adjusted R² = .60). Poorer sleep quality was independently associated with greater eveningness (MEQ: B = −0.25, SE = 0.03, β = −0.52, p <.001) and younger age (B = −0.14, SE = 0.03, β = −0.30, p <.001), while sex assigned at birth showed a small independent association (AMAB vs. AFAB: B = 1.23, SE = 0.57, β = 0.17, p = .033). Mental health-related quality of life showed only a borderline association with PSQI scores (SF-12 MCS: B = −0.06, SE = 0.03, β = −0.14, p = .063), and gender identity was not independently associated with sleep quality (binary vs. non-binary: B = −1.67, SE = 0.94, β = −0.13, p = .077). GAHT duration was not independently associated with PSQI scores (B = 0.005, p = .790). Accordingly, these findings do not support a simple linear association between longer current GAHT exposure and poorer sleep quality within this cross-sectional subgroup.

### Descriptive subgroup analyses

Participants receiving GAHT reported higher sleep disturbance compared with those not receiving GAHT (PSQI mean ± SD: 6.86 ± 3.85 vs. 5.71 ± 3.74). The prevalence of PSQI >5 was 62.0% in the GAHT group versus 48.4% in the non-GAHT group, while PSQI ≥8 was observed in 36.0% and 27.4%, respectively. Non-binary participants also showed higher PSQI scores compared with binary participants (7.97 ± 4.21 vs. 6.01 ± 3.70). The proportion of individuals scoring above PSQI >5 was 67.6% among non-binary participants and 52.7% among binary participants, while PSQI ≥8 was observed in 61.8% and 26.1%, respectively.

In multivariable analyses adjusting for age, chronotype, mental health–related quality of life, sex assigned at birth, and GAHT status, the association between gender identity and sleep quality was no longer statistically significant, whereas GAHT status remained independently associated with PSQI scores.

## Discussion

In this cross-sectional study of TGD adults attending a specialized clinical service, poor sleep quality was common but not uniform. More than half of participants scored above the conventional PSQI threshold, yet the variability of scores across the sample suggests that sleep disturbance is better conceptualized as a dimensional phenomenon than as a homogeneous characteristic of TGD populations. This interpretation is consistent with prior literature showing elevated sleep burden in transgender samples, while also highlighting marked heterogeneity in clinical presentation (7, 11).

Across analyses, chronotype and mental health-related quality of life emerged as the most consistent correlates of sleep quality. Greater eveningness was associated with poorer sleep quality in the total sample, in line with evidence linking evening chronotype to poorer subjective sleep and greater vulnerability to sleep disturbance across both psychiatric and non-clinical populations (18, 27, 28). At the same time, chronotype distribution did not differ according to sex assigned at birth, gender identity, or current GAHT status, suggesting that circadian preference in this sample is better understood as an individual characteristic rather than a group-specific feature. Lower mental health-related quality of life was also independently associated with poorer sleep quality, supporting the broader view of sleep disturbance as closely intertwined with psychological functioning (3). The association between greater eveningness and poorer sleep quality is of particular interest because it supports an individual-level, rather than group-level, interpretation of sleep vulnerability. Eveningness may increase the likelihood of circadian misalignment between endogenous preference and environmental demands, thereby contributing to worse subjective sleep and broader functional burden. In this sense, chronotype may help explain why sleep difficulties vary substantially even within clinically similar TGD individuals. The present study does not allow conclusions regarding the mechanisms underlying greater eveningness in some TGD individuals. However, previous literature suggests that sleep difficulties in sexual and gender minority populations may be related to minority stress, discrimination, psychological distress, and chronic hyperarousal, all of which may interfere with sleep regulation and perceived sleep quality (12, 13). Future studies should directly assess these factors to clarify the pathways linking circadian preference and sleep disturbance. This interpretation is also consistent with smaller transgender studies linking evening preference and insomnia symptoms to poorer mood and reduced quality of life (18).

Findings related to current GAHT status require particularly cautious interpretation. In the present study, current GAHT status was associated with poorer sleep quality and significantly moderated the association between chronotype and PSQI scores, with eveningness showing a stronger relationship with sleep disturbance among participants receiving GAHT. This pattern does not indicate that GAHT is unrelated to sleep quality; rather, it suggests that GAHT status may identify a subgroup in which circadian vulnerability is more clinically expressed. One possible explanation is biological, insofar as sex hormones may influence circadian regulation and sleep organization. However, available evidence does not support a simple or uniform effect of GAHT on sleep. Prospective studies have reported no clinically significant overall worsening of subjective sleep quality during the first year of GAHT, although small changes in insomnia symptoms and sleep parameters have been described. Likewise, objective sleep EEG studies suggest that gender-affirming hormones may be associated with selective and sex-specific changes in sleep architecture rather than broad deterioration in sleep. Against this background, the absence of an association between treatment duration and PSQI scores in our subgroup analysis argues against a straightforward dose–response interpretation. The observed GAHT × chronotype interaction may therefore reflect a more complex interplay between hormonal exposure, circadian preference, psychological burden, and contextual factors, rather than a direct treatment effect. (8, 9, 19).

Additional exploratory analyses showed that non-binary participants reported poorer sleep quality and greater eveningness than transgender men and transgender women at the descriptive level, whereas no significant differences emerged according to sex assigned at birth. However, gender identity and sex assigned at birth were not independently associated with sleep quality after adjustment for chronotype, mental health-related quality of life, age, and current GAHT status. This suggests that the higher PSQI scores observed among non-binary participants may be better understood as reflecting broader individual correlates of sleep quality rather than independent effects of identity-related variables per se. (29–31). This finding should not be interpreted as evidence that identity-related factors are unimportant. Rather, their effects may be indirect and partly conveyed through psychosocial burden, minority stress, reduced access to affirming environments, and other contextual stressors, all of which may contribute to both psychological distress and sleep disturbance. (12, 13, 32). This interpretation is consistent with emerging evidence showing that transgender and gender-diverse individuals often report poorer sleep outcomes at a descriptive level, including higher rates of sleep disturbance and shorter sleep duration. At the same time, these disparities have been linked to psychosocial stressors such as stigma, discrimination, and minority stress processes, rather than to identity categories alone. Within this framework, identity-related factors may influence sleep indirectly, through their association with psychological burden and contextual stressors, rather than exerting a direct effect. Further research is needed to clarify these pathways and to disentangle the relative contribution of social, psychological, and circadian mechanisms in shaping sleep quality in TGD populations.

From a clinical perspective, these findings support the routine assessment of sleep quality in transgender and gender-diverse individuals across psychiatric and gender care settings, particularly in those with greater eveningness and poorer mental health-related quality of life. They also suggest caution against attributing sleep complaints directly to gender-affirming hormone therapy in the absence of longitudinal evidence and more detailed treatment characterization. Rather, sleep disturbance may represent a clinically relevant marker of broader psychological and circadian vulnerability. A comprehensive clinical formulation integrating circadian preference, current psychological functioning, and contextual stressors may provide a more useful framework for assessment and intervention (12, 13, 33). This may include the integration of sleep-focused assessment and circadian-informed interventions within routine clinical care.

Several limitations should be considered in the evaluation of these results. First, the cross-sectional design precludes causal inference. Second, all measures were self-reported, including sleep quality and chronotype. Third, psychosocial stressors were not directly assessed, although they may plausibly contribute to sleep difficulties in TGD populations. Fourth, the clinical nature of the sample may limit generalizability to community-based TGD populations. Finally, treatment-related characteristics other than current GAHT status and duration were not examined. In particular, information regarding gender-affirming surgery history, gonadectomy status, hormone levels, treatment adherence, and menopausal-like symptoms was not available and may represent important factors influencing sleep quality. Moreover, the relatively short treatment duration in a substantial proportion of participants may have reduced the interpretability of duration-based analyses.

## Conclusion

In conclusion, sleep disturbance in TGD adults appears to be best understood as a multidetermined and dimensional phenomenon, primarily associated with individual circadian preference and psychological well-being rather than with identity variables alone. The association observed with current GAHT status did not extend to treatment duration and should therefore be interpreted cautiously, as potentially reflecting a more complex biological and psychosocial context rather than a simple treatment effect. Longitudinal studies incorporating psychosocial stressors, objective sleep measures, and more detailed treatment characterization are needed to clarify the mechanisms underlying sleep difficulties in TGD populations.

## Data Availability

The raw data supporting the conclusions of this article will be made available by the authors, without undue reservation.
